# A metabolic perspective of Peto's paradox and cancer

**DOI:** 10.1098/rstb.2014.0223

**Published:** 2015-07-19

**Authors:** Chi V. Dang

**Affiliations:** Abramson Cancer Center, University of Pennsylvania, Philadelphia, PA 19072, USA

**Keywords:** cancer metabolism, oxidative stress, Peto's paradox, body mass, metabolic rate

## Abstract

The frequency of cancer is postulated to be proportional to the number of cells an animal possesses, as each cell is similarly exposed to mutagens with every cell division. Larger animals result from more cell divisions with more mutagenic exposure, and hence are expected to have higher frequencies of cancer. Yet, as stipulated by Peto's paradox, larger animals do not have the higher rates of cancers seen in smaller animals despite the significant differences in cell numbers and a longer lifetime that would expose larger animals to more mutagens. The rates of cancer appear to be inversely proportional to animal body size, which scales inversely with specific metabolic rates of mammals. Studies over the past 20 years have linked oncogenes and tumour suppressors to alterations in cancer metabolism, and conversely, mutations in metabolic genes have been documented to trigger tumorigenesis. The by-products and intermediates of metabolism, such as reactive oxygen species, oxoglutarate, citrate and acetate, all have the potential to mutate and alter the genome or epigenome. On the basis of these general observations, it is proposed that metabolic rates correlate with mutagenic rates, which are higher in small animals and give the mechanistic basis for Peto's paradox. The observations discussed in this overview collectively indicate that specific metabolic rate varies inversely with body size, which seems to support the hypothesis that metabolism drives tumorigenesis and accounts for Peto's paradox.

## 1. Introduction

Activation of oncogenes and loss of tumour suppressors are mediated by DNA mutations, chromosomal instability or epigenetic alterations, thereby driving tumorigenesis [1]. The replicating genome is constantly exposed to mutagens, such as reactive oxygen species (ROS) produced from mitochondria or ultraviolet and gamma radiation from the cosmos, that damage DNA, cause imbalance in nucleotide pools that results in DNA replication stress, and alter levels of metabolites involved in modifying the epigenome (e.g. acetyl-CoA, *S*-adenosyl methionine, α-ketoglutarate) that result in misregulation of gene expression. As such, unrepaired mutagenic errors could be propagated to daughter cells and contribute to the development of cancer. It stands to reason, then, that the more cell divisions an animal has during its lifetime, the more likely it is for mutagenic events to occur in somatic cells. In fact, microsatellite alterations and genomic deep-sequencing analysis of somatic cells have been used to trace cell fates in mice, providing direct experimental evidence for the accumulation of mutations with cell division [2–5]. Hence, from this viewpoint, the larger an animal is, the more likely it should be to develop cancer during its lifetime. This supposition is, however, not support by empirical observations, which suggest that smaller animals have a relatively higher rate of cancer compared with larger animals, or conversely larger animals have a lower than relative expected rate of cancer development. In fact, quantitative analysis supports this contention [6]. This non-concordance is known as Peto's paradox [6–9].

## 2. Cell metabolism as a cause of cancer

**Figure 1.**
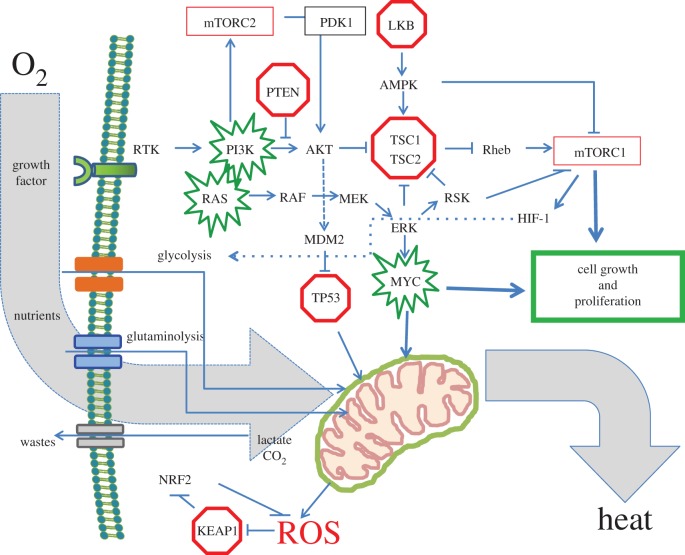
Regulation of cell metabolism by proto-oncogenes and tumour suppressors. The diagram depicts growth factor receptor activation and the cascade of events that results in nutrient uptake for biomass accumulation, energy and reductive power production. The PI3K and RAS-RAF-MEK pathways are shown signalling downstream, resulting in mTOR activation and transcriptional responses, such as the path towards Myc activation. Proto-oncogenes are highlighted in green, whereas tumour suppressors are in red. Note that the mitochondrion is a key respiratory and biosynthetic organelle, from which intermediates provide the building blocks for protein, nucleic acid and lipid synthesis. The KEAP1-NRF2 pathway is shown to be activated by KEAP1 inactivation by ROS, which is a key metabolic by-product. The overarching use of oxygen is depicted with the generation of heat and ROS.

Resting and growing cells appear to use distinct metabolic pathways, which are also different among different cell types, but share many common metabolic features [10]. The resting cell could be regarded as a bioreactor that maintains its structure and avoids entropy, as discussed by Erwin Schrödinger in his monograph, ‘What is Life?’ [11]. Metabolism, which is mediated by cellular nutrient import to generate energy and building blocks, replenishes damaged molecules and organelles to prevent entropic cellular decay (figure 1). The nutrients include glucose, glutamine, other amino acids, fatty acids and acetate [12,13]. All of these nutrients are processed through the Krebs or tricarboxylic acid (TCA) cycle to produce ATP, NADPH, lipids, nucleic acids and proteins. It is estimated that the bulk of ATP is used to maintain membrane potentials and for protein synthesis [14,15]. A cell that is stimulated to proliferate uses nutrients to produce ATP, NADPH for reductive biosynthesis and building blocks to make a copy of itself.

Resting cells tend to use oxidative phosphorylation, which efficiently oxidizes acetyl-CoA for ATP production. In many instances, resting cells can use fatty acids which are the richest energetic nutrients that can provide up to 129 ATP molecules per molecule of palmitate (e.g. [16]). The TCA cycle comprises a series of reactions that oxidize acetyl-CoA through several dehydrogenases that are coupled with the conversion of NAD^+^ to NADH, which store high energy electrons [16]. These electrons are channelled through the electron transport chain in mitochondria to create a proton gradient across the inner mitochondrial membrane that drives a chemical energy gradient to generate ATP from ADP.

The canonical replicating cell uses both glucose and glutamine as major nutrients. Glucose is a vital nutrient through which plants store sunlight energy and animals use as a central bioenergetic currency that is regulated by a sophisticated hormonal system, balanced by insulin and glucagon [16]. Glucose imported into cells is subsequently converted via glycolysis to pyruvate. Pyruvate is a vital metabolite central to several pathways that produce alanine through transamination, lactate through reduction, oxaloacetate through carboxylation or acetyl-CoA through dehydrogenation. Pyruvate could be oxidized to acetyl-CoA by growing cells or used to regenerate NAD^+^ from NADH through its conversion to lactate.

Glutamine is another critical nutrient for growing cells, which import glutamine and convert it to glutamate by glutaminase [17–20]. Glutamate is converted by either glutamate dehydrogenase or transaminases to α-ketoglutarate, which is further oxidized in the TCA cycle as described above. Glutamine circulates at the highest concentration (0.5 mM) among amino acids in humans, and glucose level is tightly controlled by hormones, such that our cells as constantly bathed in these energy sources [21]. However, normal cells do not proliferate upon exposure to glucose or glutamine, as does the unicellular yeast cell, but rather they require an additional signal—growth factors that engage receptors (figure 1). Upon activation of a growth factor receptor, signal transduction pathways activate mTOR to sustain the post-translational signalling of growth by increasing protein synthesis. Signalling pathways also activate transcriptional programmes in the nucleus, which produce mRNAs that are essential for ribosome biogenesis, protein, nucleic acid, lipid and carbohydrate synthesis [13,22,23]. In essence, the stimulated cell is a biochemical reaction vessel that drives nutrients into the vessel or cell for the production of daughter cells with high-fidelity replicated genomes, consuming oxygen and producing heat and ROS (figure 1).

Metabolic processes of a growing cell generate by-products that could be toxic to cells, and hence systems to neutralize these toxins have evolved. Lactic acid is exported by monocarboxylate transporters, while carbon dioxide can be eliminated by carbonic anhydrase [24–27]. ROS, which can be highly toxic, are neutralized acutely by glutathione or peroxiredoxins and subacutely by an anti-oxidant transcriptional response through the KEAP1-NRF2 system [28,29]. KEAP1, an inhibitor of NRF2, has uniquely sensitive sulfhydryl groups that can be oxidized by ROS, rendering KEAP1 inactive. Inactivation of KEAP1 by ROS activates NRF2 (figure 1). NRF2 is a transcriptional factor that induces the transcription of genes for the production of ROS-neutralizing enzymes such as superoxide dismutase, which converts superoxide to hydrogen peroxide, or catalase, which converts hydrogen peroxide to oxygen and water. Thus, growing cells need anti-oxidant mechanisms to protect them from the toxic and mutagenic effects of ROS.

During development and in normal adult tissues, there are anatomical regions within tissues that have diminished oxygen levels or hypoxia, which reprogrammes cellular metabolism through the hypoxia-inducible factors (HIFs) [30–33]. HIF-1 is documented to transcriptionally activate genes involved in glycolysis or the conversion of glucose to pyruvate and subsequently to lactate through lactate dehydrogenase (figure 1). HIF-1 also increases the importation of glucose by driving the expression of glucose transporters. With oxygen deprivation, pyruvate is diverted away from acetyl-CoA or the TCA cycle through induction of pyruvate dehydrogenase kinase by HIF-1 and shunted to lactate through activation of lactate dehydrogenase A by HIF-1. In this manner, hypoxic cells can survive by means of anaerobic glycolysis. Recent studies have also revealed that certain cells can backfill the hypoxic TCA cycle with glutamine-derived TCA cycle intermediates to enable cellular survival.

Studies of basic cell metabolism have taught us the general lessons that resting cells tend to use oxidative phosphorylation, whereas proliferating cells use both oxidative phosphorylation and aerobic glycolysis for the conversion of glucose and glutamine to ATP and the metabolic intermediates required for building key components of a cell. Production of ROS by the mitochondria, peroxisomes and also from the NADPH oxidases generates mutagens that could have driven genomic diversity during evolution over the past billion years. Indeed, the acquisition of mitochondria through endosymbiosis is believed to have been pivotal for the evolution of complex organisms, which presumably have arisen from rapid genomic diversity through bioenergetics, mutations and subsequent natural selection [34,35]. In similar fashion, cancer can result from endogenous mechanisms of mutagenesis via ROS that can activate oncogenes and cause the loss of tumour suppressors. Thus, metabolism appears to be able to cause mutations, driving the development and evolution of cancer.

The supposition that metabolism can cause cancer is further underscored by studies of familial cancer syndromes that are linked to mutations in metabolic enzymes [36,37]. Mutations in succinate dehydrogenase (SDH) subunits are linked to familial syndromes of paraganglioma or pheochromocytoma. Fumarate hydratase (FH) mutations are associated with heritable predisposition to leiomyoma, leiomyosarcoma or renal cell carcinoma. More recently, somatic mutations of isocitrate dehydrogenase 1 or 2 have been found frequently in gliomas, acute myelogenous leukaemia, angioimmunoblastic lymphoma and chondrosarcoma [38]. These mutations occur in enzymes that are either upstream (isocitrate dehydrogenase 2, IDH2) or downstream (SDH or FH) of the key TCA cycle metabolite α-ketoglutarate, which also serves as a cofactor for many dioxygenases. In the case of IDH, the mutant neo-enzyme converts α-ketoglutarate to 2-hydroxyglutarate, which can inhibit dioxygenases. With SDH or FH mutations, the accumulation of succinate or fumarate, respectively, can also inhibit dioxygenase activities. These dioxygenases included the prolyl hydroxylase domain (PHD) enzymes, which mediate HIF degradation, and oxygenases that are involved in epigenetic regulation via their DNA (TET) or histone (jumonji proteins) demethylase activities [39,40]. In this regard, it has been documented that acute myeloid leukaemia (AML) or glioma tumours with IDH mutations tend to cluster into groups with distinct genome-wide methylation patterns [38,41]. These observations suggest that these mutations alter metabolism, which in turn changes the epigenome that predisposes to tumorigenesis. As such, mutations in metabolic enzymes provide evidence for metabolism as a cause of cancer.

Another factor that contributes to tumorigenesis is inflammation which results from injury or infectious agents [42]. In this regard, it is notable that many of the more common cancers are associated with organs with more immediate exposure to the external world, such as the lung, breast, prostate and gastrointestinal tract. In fact, the gastrointestinal tract, which has its own immune defence hubs or Peyer's patches comprising lymphoid cells, is teaming with the gut microbiota, which is emerging as a key factor in health and disease, particularly cancer [43]. The gut microbiota and its metabolism are documented to contribute to disease, such as the association of a pro-atherogenic compound, trimethylamine-*N*-oxide, with cardiovascular disease. It is probable that inflammatory metabolites from the microbiota stemming from short chain fatty acids could well contribute to tumorigenesis. Thus, whether the microbiota plays a role in Peto's paradox is unknown, but it is a factor that should be considered.

## 3. Metabolism and cancer rates in animals

If metabolism can drive tumorigenesis through ROS and imbalances in metabolites such as nucleotide pools, then the rate of metabolism could correlate with the rate of cancers among animals of different sizes. The correlation between animal size and cancer rates, particularly for large animals, has not been solidly established as studies among very large animals are very limited [7,8]. For example, it is cited in the literature that whales and elephants rarely are found with cancers, whereas feral mice have lifetime cancer rates of up to 50%, but the primary literature supporting this cannot be easily found. A comprehensive review on marine mammal cancers cited studies with relatively significant numbers of necropsies, and the data suggest that whales have much lower cancer rates relative to smaller mammals, such as the sea lion [44]. An inspection of a worldwide database of over 15 000 wild or captured elephants appears to support the claims of Peto's paradox. Of the 616 deceased elephants in this database (http://www.elephant.se), 18 (approx. 3%) are documented to be associated with cancer; however, these numbers do not provide a lifetime rate of cancers. Nonetheless, these data suggest that elephants do not often die because of cancers, compared with 12.5% of human deaths attributable to cancer among all causes. Natural murine deaths with aging have been documented for a group of over 2000 animals, of which about 50% succumbed to cancer by 800 days of age [45]. These observations appear to support but do not prove the general contention of Peto's paradox.

**Figure 2.**
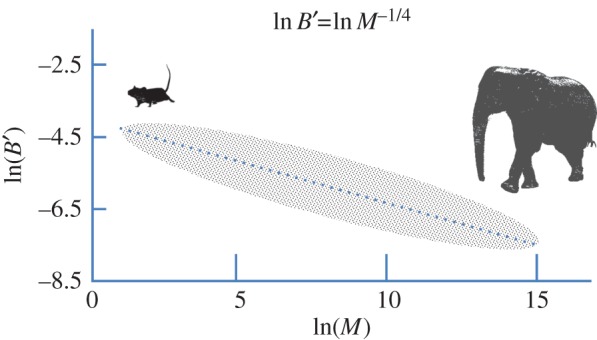
Idealized representation of Klieber's law. The diagram demonstrates the relationship between specific metabolic rates (*B*′) (W g^−1^) and body mass (g) as a log–log plot. Experimental data point scatter is depicted by the stippled oval. The equation shown provides a slope of −1/4, which could vary within any range of body masses. More refined fitting of empirical data suggests a curved rather than a linear relation between ln*B*′ and ln*M*. (Online version in colour.)

If one were to assume that it is true that larger animals have relatively lower rates of cancer burden compared with smaller mammals, could metabolic rates that scale with body size be associated with the propensity for tumorigenesis? The correlation of animal body size with basal metabolic rates has been recognized for many decades, starting with the observations of Max Klieber in the 1930s which document a relationship between animal body mass and the amount of heat production per day [46] who found that body mass best correlated with 3/4 power of whole body basal metabolic rate (*B*) (*B* = *M*^3/4^; where *M* is body mass). This power law is known as Klieber's law, which has been a matter of debate regarding the exact magnitude of that power; i.e. 2/3 versus 3/4. It had been argued that basal metabolic rate relates to heat loss through body surface area, which would be more closely aligned with the 2/3 power. An updated examination of extant data suggests that the power function is closer to 3/4 than 2/3, although there is significant variability in subgroups of mammals. It is notable that mass-specific metabolic rate *B*′ is defined as *B*/*M* and reflects metabolic rates normalized to tissue mass, such that *B*′ = *M*^−1/4^ (figure 2): when plotted as log*B*′ versus log*M*, the slope (approx. −1/4) is the exponent. From a biological perspective, this power function is observable from the significantly higher metabolic rates of mice, whose rates are orders of magnitude higher than that of elephants [47]. Further, this is reflected in the significantly different amounts of food consumption in mice (approx. 20% body mass of food per day) compared with elephants (approx. 5% body mass per day). Specifically, Klieber estimated that one steer weighing the same as 300 rabbits would take 120 days to eat 1 ton of hay, while 300 rabbits would finish 1 ton of hay in 30 days [48]. What hypothesis then would account for the power function relating *B* or *B*′ to *M*?

**Figure 3.**
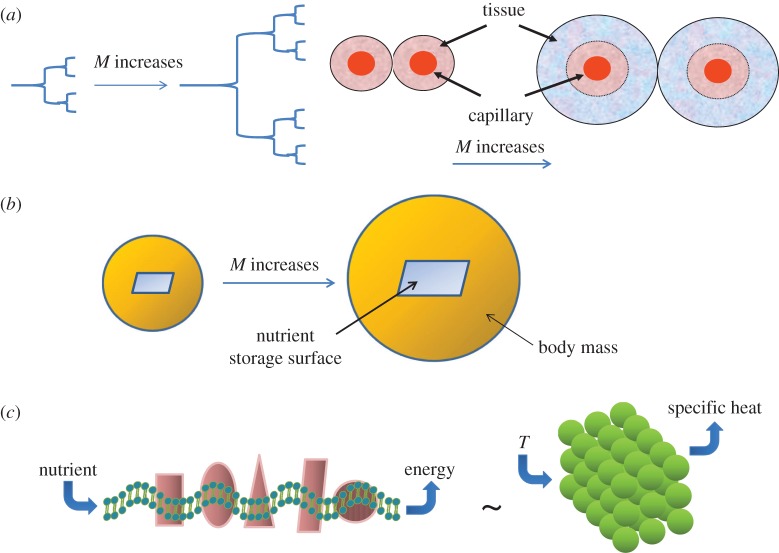
Metabolic theories for Kleiber's law *B* = *M*^3/4^. (*a*) The WBE model assumes that metabolic rates are determined by the geometry of the nutrient delivery, which is modelled as a fractal with more sequential branchings of the vasculature in larger animals (*a*, left diagram). Higher branching results in larger distances between capillaries that lead to decreased metabolism and perfusion to more hypoxic (blue areas in the right diagram) tissues in larger animals. (*b*) The DEB model is based on the concept that metabolic rates depend on access to energy depot, which is assumed to be related to body surface area. With the DEB theory, the body mass is related to metabolic rate with a 2/3 power function that approaches the 3/4 power as body mass approaches infinity. (*c*) The quantum metabolism theory is based on the concept that bioenergetic (mitochondrial) membranes store energy as coupled oscillators (conceptually depicted by the wavy membrane) whose energy output depends on the nutrient delivery rate and the enzymatic oscillatory cycle time. The model relies (depicted as ∼) on the theoretical foundation of Einstein–Debye to derive the relation between temperature (*T*) and specific heats of solids based on quantized elastic waves of solids (illustrated on the right). This theory accounts for the variations in the power function observed empirically.

A theoretical basis for the 3/4 power law function was proposed by West, Brown and Enquist (WBE), who focused on nutrient delivery through the geometry of the circulatory system as the cause of the power law [49–52] (figure 3*a*). In essence, these researchers investigated a theoretical model (termed the WBE model) of the circulatory system, which resembles a fractal network originating from the central aorta branching all the way down to the end capillaries. If one assumes that the branching pattern is repeated from aorta to capillaries, then the end capillary density in this model would predict the rate of perfusion of nutrients to cells, which determines metabolic rate. This model predicts that the larger the animal, the sparser is the capillary density in tissues. The larger inter-capillary distance is thought to cause decreased delivery of nutrients and a steeper oxygen gradient from blood vessels, causing decreased respiration and oxidative phosphorylation, and culminating in diminished specific metabolic rates (figure 3*a*). Intriguingly, this theoretical framework resulted in a 3/4 power function relation between *M* and *B*. This theory does not account for the variation in the power function (2/3 to 1) and assumes a cell-independent cause for the power relation based solely on nutrient delivery that depends on the fractal geometry of the circulatory system. In fact, a closer examination of the extant data reveals a curvature of the relation between log*B* and log*M*, which is not strictly linear and does not fit the WBE model [53]. Modifications of the WBE model, however, could improve the theoretical model fit with empirical data. This work suggests that the general concept of nutrient resource distribution could provide the mechanistic underpinning for the scaling of metabolic rates with body mass.

Although the WBE theoretical framework appears compelling and fits the observed power law function of *B* = *M*^3/4^, the evidence for alterations in capillary density as a function of animal body size is not well documented. In fact, examination of the literature on capillary density in key muscle tissues does not fully support this contention [54]. Schmidt-Nielsen & Pennycuik [55] compared the capillary density across 10 mammals by studying masseter and gastrocnemius muscles. While the smallest of mammals appear to have some of the highest capillary densities, such as those found in bat and mouse, capillary density does not scale monotonically over larger animals. It appears that other factors, such as the type of muscle, activity and cold acclimation, may play a significant role in determining capillary density. Activity appears to be a key factor in capillary density and perfusion, as capillary blood transit time (a measure of tissue perfusion) correlates with muscle mitochondrial density and intrinsic activity of specific animals. For example, the dog body mass is roughly equivalent to those of goats, but goats being less active have a twofold lower mitochondrial volume density. Another study by Kayar *et al*. [56] examined capillary and mitochondrial volume density in muscles of five mammals with body masses ranging from 3 to 447 kg. Capillary density did not scale with body mass, and the mitochondrial density appears highest in the most active animals, correlating with muscle–mass-specific maximal oxygen consumption. However, body mass scales best with mean transit time for blood in muscle capillaries at maximal aerobic exercise, suggesting that perfusion rather than capillary density may be important for the scaling of metabolic rates with body mass. Hence, experimental data only partially support the WBE model at extremes of body masses, for which capillary density correlates with body mass in one study. Although these studies are only limited to muscle groups and did not extend to other organs, these observations question the validity of the elegant WBE model, which assumes a fractal branching network of the vasculature as the basis for the power law function relation between *B* and *M*. Elegance of a theory, therefore, may not reflect reality.

In contrast to the WBE model, the dynamic energy budget (DEB) theory suggests that scaling of energy storage depot and body mass could underlie the scaling relationship of *B* = *M*^3/4^ [54,57–59] (figure 3*b*). This model assumes that animal energy storage depot scales with surface area, because it is the surface area of depot that would be available for organisms to access stored nutrients. Access to the energy is assumed to drive the metabolic rate, particularly since animals have prolonged non-feeding periods and hence depend on stored energy. This assumption leads to scaling of the depot surface to body mass with a 2/3 power, but when body mass approaches infinity the exponent approaches the Kleiber power of 3/4. Experimental data to support the DEB theory are sparse; however, if one assumes that small intestine surface area is representative of surface area of nutrient depot, then a 2/3 power function relating to body mass is found [54].

Another theory, termed quantum metabolism, focuses on the presumed quantum behaviour of cell-dependent membrane bioenergetics properties as the basis for the power law [60,61] (figure 3*c*). The theory is based on coupled energy-transducing oscillator networks confined to a specified space, integrating chemi-osmosis and the quantum thermal properties of solids. Demetrius proposed that bioenergetic membranes, such as those of the mitochondrion (figure 3*c*), store and generate energy in the membrane as coupled molecular oscillators. These confined oscillators resemble the coupled molecular vibrations of solids, whose thermal properties could be derived by treating the coupled oscillations as quantized elastic waves according to the Einstein–Debye derivation of the heat properties of solids. The specific heat of solids, for example, could be related to absolute temperature. Further, the quantum metabolism theory accounts for the enzymatic turnover or cycle time of the membrane-bound respiratory chain that stores and produces energy as a function of the time of nutrient flow. A scaling law relating body mass and metabolic rate with a power of 3/4 was hence found when the dimensionality (3D) of physical space and the mean oscillatory cycle time in mammals were included. A change in dimensionality can alter the power exponent, varying from allometric (less than unity) to isometric (unity).

Some cell-dependent factor could then theoretically contribute to the curvature of the log*B*′ versus log*M* function (figure 2). The quantum metabolism model suggests a cell-dependent component and contrasts with the other models that focus primarily on energy supply. Experimental data supporting cell-dependent differences appear conflicting. In one study of isolated liver cells from mammals with body masses varying from 0.02 g to 200 kg, the oxygen consumption rates of hepatocytes appear to vary, with the highest rates being found in smaller animals [62]. In fact, electron micrographs indicate that there are fewer mitochondria in cells of a larger animal compared with a smaller one, but mitochondrial density could not fully account for the differences in cellular metabolic rates. While these observations support the quantum metabolism theory, other studies of primary skin fibroblasts and skeletal muscle from various mammals did not reveal the scaling with body mass that was seen in liver cells [63,64]. It is notable, however, that skin cells could use oxygen directly from the air rather than from the circulation, but whether this accounts for the differences between skin versus liver cells is unknown. Another study of muscle enzymes suggests that oxidative enzymes scale inversely with body mass, whereas glycolytic enzyme activities scale proportionally with body mass [65]. The scaling of enzymes suggests cellular adaptation to nutrient delivery according to the WBE model, but supports the idea of a cell-dependent basis for metabolic scaling according to the quantum metabolism theory. Overall, the extant evidence points to detectable scaling of cell-dependent metabolic rates with body mass. However, given the uncertainties and differences in observations, further experimentation is necessary to determine whether there are true cell-dependent differences in metabolic rates as a function of body size.

Although the exact underlying mechanistic basis for the observe power law function is unknown experimentally, these three theoretical frameworks are based on metabolic concepts and provide putative insights into mechanisms. Empirically, it has been observed that mammalian sleep time scales with body mass, particularly for herbivores [66]. Brain-specific metabolic rates scale inversely with body mass. As such, the size of the brain, which consumes significant energy in proportion to body mass, is also inversely related to sleep time, such that smaller animals sleep much longer than larger ones. The vole, for example sleeps on average approximately 12 h d^−1^ versus the elephant that sleeps about 4 h d^−1^. Experiments with sleep deprivation in the rat documented ROS-induced damage to brain cells, indicating that sleep is required to diminish metabolism and allow time for repair [67,68]. Hence, it appears that higher metabolic rates in the brain are associated with longer periods of sleep to repair ROS-induced damage incurred by the biochemical stress of waking metabolism. Recently, it was documented that sleep is associated with a 60% increase in the brain interstitial space allowing for convection of cerebrospinal fluid (CSF) and interstitial fluid to clear neurotoxic metabolites that presumably accumulated during waking time [69]. This study is corroborated by a human sleep deprivation study of CSF levels of amyloid protein, revealing that sleep-deprived normal subjects have higher levels of amyloid protein in their CSF [70]. These observations collectively account for an inverse relationship between body mass and sleep time, and further support the idea the higher metabolic rates are associated with higher ROS and metabolic stress that can lead to tumorigenesis.

Based on concepts similar to the WBE model for whole body metabolic rates, Herman *et al*. [71] proposed a quantitative theory of solid tumour growth, vascularization and metabolism. One conclusion drawn from this theory is that in a mouse host human and mouse tumours are expected to grow at similar rates, in contrast to markedly slower tumour growth rates in humans, which have slower nutrient perfusion rates through the cancer tissue. This theory also accounts for Peto's paradox, arguing that the power density driving mutagenic biochemical reactions (such as ROS production) within a cell scales as *B*_c_ ∼ *M*^−1/4^. Hence, overall cancer incidence would scale as *M*^−1/4^ log*M*, which is dominated by *M*^−1/4^. This would then lead to the prediction that cancer incidence scales inversely to maximum lifespan, which scales approximately as body size to the 1/4 power (*M*^1/4^). Thus, smaller animals would be expected to have a greater incidence of cancer than larger mammals.

## 4. Metabolism, ageing and tumorigenesis

Although no specific experiments could be designed to test directly the hypothesis that Peto's paradox is based on scaling of metabolic rates as a function of body mass, experiments that have been performed to slow down metabolism appear to provide some supportive evidence for a role of metabolism in tumorigenesis. In fact, processes that curb metabolism appear to prolong the lifespan of experimental animals and diminish cancer incidence [72]. Caloric restriction from yeast to mice has been clearly linked to prolongation of lifespan and decreased tumorigenesis in certain cancer models [73]. Although the basis for the effects of caloric restriction in cancer is complex, an effect on cellular metabolism has been implicated. Inhibition of mTOR through the use of rapamycin or genetic deletion is associated with prolonged lifespan in mice [74,75]. It stands to reason that since mTOR is essential for anabolic metabolism, diminished mTOR signalling would decrease cellular energy expenditure and tumorigenesis [75]. Indeed, genetic knockout of mTOR is associated with decreased formation of spontaneous cancers in mice [74]. Similarly, rapamycin also diminishes spontaneous tumorigenesis [76]. Presumably, decreased energetic demands for cell growth through mTOR inhibition should also decrease demands on mitochondrial output. As such, diminished mitochondrial activity would be expected to prolong survival and decrease tumorigenesis. The anti-diabetic drug metformin is an inhibitor of mitochondrial activity via inhibiting mitochondrial respiratory complex I. It has been observed that metformin can prolong the lifespan of mice and reduce both spontaneous and genetically engineered cancers in mice [77,78]. These observations, collectively, suggest that diminished mitochondrial output or function can prolong lifespan and reduce tumorigenesis. Although these studies do not address Peto's paradox directly, the fact that diminished metabolism protects again tumorigenesis is consistent with a role of metabolism in the rate of cancer incidence in animals.

## 5. Concluding remarks

In this essay, fundamental aspects of cancer cell metabolism are reviewed and evidence in support of a metabolic basis for Peto's paradox is discussed. There is compelling evidence that metabolic rates (*B*) scale with body mass (*M*) in mammals with an approximate power function: *B* = *M*^3/4^ (or ln*B*′ = ln*M*^−1/4^), for which theoretical models have been proposed that derive this power function from metabolic concepts that pertain to variations dictated by animal body size. The relationship between metabolic rates and cancer incidence appears to be supported by evidence that suggests metabolism drives tumorigenesis, which could be curbed by decreased metabolic demands through treatment of animals with mTOR inhibitors or by inhibition of mitochondrial respiration. Collectively, this body of evidence supports the hypothesis that metabolism is a vital factor in Peto's paradox.
